# Observations on the Use of Arsenic in Intermittent Fevers

**Published:** 1787

**Authors:** Robert Willan

**Affiliations:** Member of the Royal College of Physicians, and Physician to the Finsbury and Public Dispensaries in London


### XIV. *Obſervations on the Uſe of Arſenic in Intermittent Fevers. By* Robert Willan, *M. D. Member of the Royal College of Phyſicians, and Phyſician to the Finſbury and Public Diſpenſaries in London.*

IN a pamphlet lately publiſhed by Dr. Fowler, Phyſician at Stafford, arſenic has been recommended as a ſafe and effectual remedy for the cure of intermittents *. This mode of practice was at firſt exclaimed againſt by the cautious part of the faculty, from an apprehenſion that the indiſcriminate uſe of ſo active a mineral might be productive of dangerous conſequences. Several practitioners, however, ventured to adminiſter it, and gave a favourable report of their ſucceſs. I was induced, at Dr. Fowler's requeſt, to make an extenſive trial of its powers in many obſtinate agues which came under my care during the preſent ſpring, and for that purpoſe had ſome of the ſolution made according to the formula preſcribed in the treatiſe above mentioned. The account of my experience on this ſubject ſeems to be not improper for the Medical Journal.

* See Vol. VII. page 192.

CASE

### CASE I.

John Hawes, aged twenty-ſix years, was admitted at the Public Diſpenſary, in March, 1787, for a tertian intermittent, which had been ſuffered to go on ſome weeks without any proper treatment. The diſorder not yielding to the firſt exhibition of the bark, I ordered (after an emetic) twelve drops * of the mineral ſolution to be taken three times a day in barley water. The fit never returned afterwards, and he was diſmiſſed at the end of a fortnight in perfect health. The above doſe did not produce nauſea, griping, or any other diſagreeable ſenſation.

### CASE II.

A young woman, about ſeventeen years of age, admitted at the ſame time, had laboured under a tertian ague for nine weeks. An emetic and purgative adminiſtered at the beginning of her complaint, and afterwards the bark, had produced no effect.

March 17th, ſhe began to take fifteen drops of the ſolution thrice a day, and underwent the

* Eighty drops of the ſolution contain about half a grain of arſenic. — See Vol. VII. page 197.

next fit in courfe; but had after that no more. The medicine was continued for ten days without any fenfible operation.

### CASE III.

Charles Deves, twelve years old, of a delicate and irritable habit, and confiderably emaciated, was taken in April, 1787, with regular paroxyfms of a quotidian. As there feemed to be a tendency to abdominal congeftions, I thought it material to ftop the progrefs of the difeafe early. After the proper evacuations, finding his ftomach would not bear Peruvian bark, bitters, or other tonics ufually employed, I ordered ten drops of the folution to be given as in the former cafes, and was pleafed to obferve an equal effect take place immediately. It prevented the return of the fits, without occafioning any bad fymptom, notwithftanding the difagreeable circumftances of this patient. His conftitution was in a fhort time fo much improved under the continued ufe of the folution, that he undertook a place of fome labour, where he yet remains, and in good health.

### CASE IV.

George Egintown, aged thirty-fix, for a quotidian of three weeks ftanding, was directed, April the 21ft, 1787, to take fifteen drops of the folution, as ufual. He increafed the dofe of his own accord to twenty-feven drops, which made him very fick. On the 25th I found that his fit had never returned. He complained of great pain and ftiffnefs in his arms and fhoulders, which he attributed to the medicine. This fymptom prefently abated, he being difmiffed, cured, on the 30th.

### CASE V.

John Shepherd, aged forty years, after a catarrhal complaint, was attacked with irregular fhiverings, which returned at firft every day; afterwards every third day. Thefe continued generally two or three hours at a time, and were fucceeded by profufe fweating throughout the night. Emetics, bark, and elixir of vitriol, had afforded him no alleviation. From April the 11th to April the 23d, he took the folution in dofes of fifteen drops, and never felt any return of the fhivering fits or fweating.

He

He was difmiffed a few days after in good health and fpirits.

### CASE VI.

In the beginning of April Mr. Champney, Apothecary in Holborn, mentioned to me a cafe of obftinate tertian in St. Andrew's workhoufe, which had for nine weeks refifted every plan of treatment. The patient was a woman, aged forty, of a debile conftitution, and accuftomed to little exercife. Emetics, bark, joined with pulv. rad. ferpentar. and zinzib. alum, and fal. tartar. vitriolum album, &c. properly applied, had failed of producing the defired effect.

I requefted Mr. Champney to make trial of the arfenical folution, in the dofe of twelve drops, to be gradually increafed to twenty. The return of the next expected paroxyfm was prevented by this plan; but the complaint afterwards recurred twice, and then difappeared, leaving her to recover ftrength gradually. If ever fhe took more than fifteen drops of the folution, it made her a little fick. I called upon this patient on the 11th of April, when fhe complained of pains in the arm and fhoulders, as in the cafe of George Egintown. Thefe pains went away, even while the medicine was continued in a moderate dofe.

The weather being at the time remarkably cold, I was doubtful whether to refer this fymptom, in thefe two cafes, to the remedy, or to the feafon: it did not occur on any other occafion.

### CASE VII.

A young man, about twenty-feven years of age, being cured of a tertian ague by the three firft dofes of the folution, difcontinued his attendance. Some time after, having incautioufly expofed himfelf to cold, he fuffered a relapfe, and, of his own accord, began to take the remedy again, but without making any impreffion upon his diforder. He therefore applied to me once more; and it gave me pleafure to obferve, when the catarrhal complaints were fully removed, that his paroxyfms were immediately prevented by the ufe of the folution.

The above cafes I have given in detail, as being the firft which occurred, and thence foliciting more particular attention. It feems only neceffary farther to add a general report from the fum total of patients treated in this manner. The folution was prefcribed for about forty others, others, in different ſpecies of intermittents, and ſucceeded almoſt inſtantaneouſly in every caſe.

I gave it at different ages, from five years to ſeventy-two, in doſes proportionable, viz. from four drops to twenty. No nauſea, pains of the ſtomach, or griping, were occaſioned by it, except in the two patients before mentioned. In ſhort, I do not know a medicine more ſafe than the arſenical ſolution, when it is thus cautiouſly adminiſtered, nor any one that anſwers the end propoſed more pleaſantly and effectually.

I can alſo add the teſtimony of ſeveral gentlemen to the account now delivered; I may particularly mention Mr. Bell, Surgeon at Cheſter le Street; Dr. Marſh, of Taviſtock Street, Bedford Square; and my colleague, Mr. Pearſon, Surgeon to the Public Diſpenſary, and to the Lock Hoſpital.

A medicine of no diſagreeable ſenſible qualities, and which, in a very ſmall bulk, produces effects ſo conſiderable, muſt be looked on as an acquiſition to medical practice. It is valuable, as a cheap and ſure remedy for the moſt obſtinate caſes of intermittent fevers, and eſpecially in children, who will ſeldom bear the bark, or other medicines commonly employed.

ployed. Its ufe may be farther extended to a variety of difeafes, as we now know the mode of adminiftering it fafely. I have myfelf tried it in fome particular diforders with fatisfaction, but am not yet prepared to write fpecifically on them. My fuccefs in the cafes above defcribed occafioned an agreeable furprife, and has entirely done away the fufpicions I at firft entertained refpecting the internal ufe of arfenic. Dr. Fowler, by ftanding forth to recommend a more general, though cautious, adminiftration of this mineral, has been expofed to fome degree of obloquy. I am induced to publifh thefe few obfervations, not only with a view of doing juftice to my friend, but from thinking the fubject of importance to the faculty at large. The principal objection made to this remedy, from the mifchievous confequences it may be fuppofed to produce when applied by the ignorant and unfkilful, will, I apprehend, have little weight with philofophical inquirers, whofe wifh is to promote medical fcience.

*Ely Place, Holborn,*

May 30th, 1787.

CATA-

